# NiH-catalyzed C–N bond formation: insights and advancements in hydroamination of unsaturated hydrocarbons

**DOI:** 10.1039/d3sc05589b

**Published:** 2023-12-06

**Authors:** Changseok Lee, Hyung-Joon Kang, Sungwoo Hong

**Affiliations:** a Center for Catalytic Hydrocarbon Functionalizations, Institute for Basic Science (IBS) Daejeon 34141 Korea hongorg@kaist.ac.kr; b Department of Chemistry, Korea Advanced Institute of Science and Technology (KAIST) Daejeon 34141 Korea

## Abstract

The formation of C–N bonds is a fundamental aspect of organic synthesis, and hydroamination has emerged as a pivotal strategy for the synthesis of essential amine derivatives. In recent years, there has been a surge of interest in metal hydride-catalyzed hydroamination reactions of common alkenes and alkynes. This method avoids the need for stoichiometric organometallic reagents and overcomes problems associated with specific organometallic compounds that may impact functional group compatibility. Notably, recent developments have brought to the forefront olefinic hydroamination and hydroamidation reactions facilitated by nickel hydride (NiH) catalysis. The inclusion of suitable chiral ligands has paved the way for the realization of asymmetric hydroamination reactions in the realm of olefins. This review aims to provide an in-depth exploration of the latest achievements in C–N bond formation through intermolecular hydroamination catalyzed by nickel hydrides. Leveraging this innovative approach, a diverse range of alkene and alkyne substrates can be efficiently transformed into value-added compounds enriched with C–N bonds. The intricacies of C–N bond formation are succinctly elucidated, offering a concise overview of the underlying reaction mechanisms. It is our aspiration that this comprehensive review will stimulate further progress in NiH-catalytic techniques, fine-tune reaction systems, drive innovation in catalyst design, and foster a deeper understanding of the underlying mechanisms.

## Abstract 2

This review highlights recent progress in NiH-catalyzed hydroamination, focusing on its application to a variety of alkenes and alkynes.

## Graphical abstract

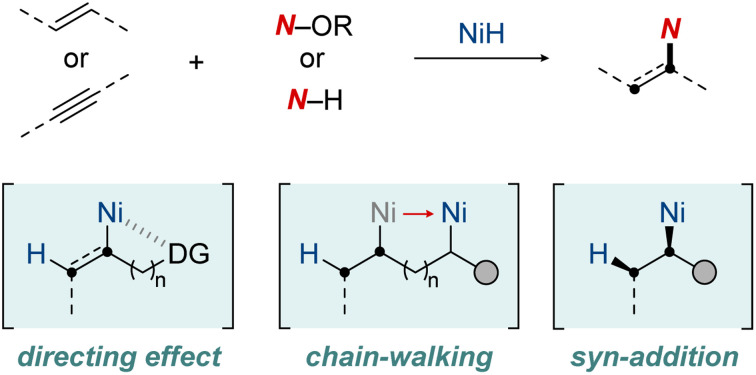

## 1. Introduction

The formation of carbon–nitrogen (C–N) bonds holds a central position in organic synthesis, paving the way for the production of diverse, biologically relevant molecules.^1–17^ Among the various techniques available for C–N bond formation, hydroamination and hydroamidation reactions have emerged as remarkably versatile and robust strategies.^18–25^ This process involves the addition of amines or their derivatives to unsaturated carbon–carbon (C–C) bonds, affording essential building blocks efficiently.^26–31^ Due to its ability to selectively introduce nitrogen functionalities into organic molecules, hydroamination and hydroamidation reactions find applications across diverse fields, from pharmaceuticals to materials science.^32–42^ Traditional approaches, which involve the nucleophilic reaction of amines with unsaturated substrates, often require challenging conditions due to the electron-rich nature of both reactants. Consequently, these methods often require harsh conditions, such as excessive amounts of substrate or elevated temperatures.^43–45^ As an innovative alternative, the pioneering work of the Buchwald and Miura groups introduced CuH-catalyzed hydroamination.^46,47^ This method works through a mechanism where the polarity is reversed: the hydrogen atom is derived from a hydridic reagent, while the amino group comes from an electrophilic reagent. By separating the R_2_N and H components into distinct high-energy reagents, the conventional hydroamination process can be altered to become a notably exothermic reaction, providing a substantial driving force.

The rise of NiH catalysis has notably shifted the landscape of this field.^48–59^ While nickel has historically been valued as a competitive alternative to precious metals like palladium and platinum due to its catalytic properties, recent applications have elevated its significance beyond being merely cost-effective. The comparative analysis of CuH and NiH in hydroamination and hydroamidation reactions reveals their unique chemical properties and reactivities. While both CuH and NiH find applications in these reactions, their distinct properties often dictate their suitability for specific substrates and reaction conditions. NiH, with its compatibility with sensitive functional groups and ability to perform chain-walking, emerges as a more adaptable candidate for complex organic syntheses. Specifically, the three distinctive differences between CuH and NiH catalysis can be described as follows: (1) coordination with directing groups: nickel's propensity to easily coordinate with directing groups stands out as a notable advantage. This coordination ability allows for the regioselective functionalization of olefins. Such precision is invaluable in the synthesis of complex molecules where regioselectivity is paramount. (2) Chain-walking ability: CuH species typically lack chain-walking capability, confining their reactivity to the immediate vicinity of the metal center. This limitation often restricts the functionalization to positions adjacent to the metal. In contrast, NiH species, when combined with suitable chain-walking ligands, can engage in subsequent cross-coupling at distal positions. This chain-walking feature imparts the ability to perform regioselective functionalization of both remote and proximal olefins, significantly enhancing the versatility in organic molecule modification. (3) Compatibility with functional groups: CuH species are known for their strong reductive nature. While this reductive quality is a key feature, it can also restrict their compatibility with substrates containing sensitive functional groups such as ketones and aldehydes. Consequently, this may limit the variety of substrates suitable for hydroamination and hydroamidation reactions. On the other hand, NiH species show an enhanced compatibility with these sensitive groups, allowing for a wider range of substrates to be utilized in hydroamination and hydroamidation reactions.

**Scheme 1:**
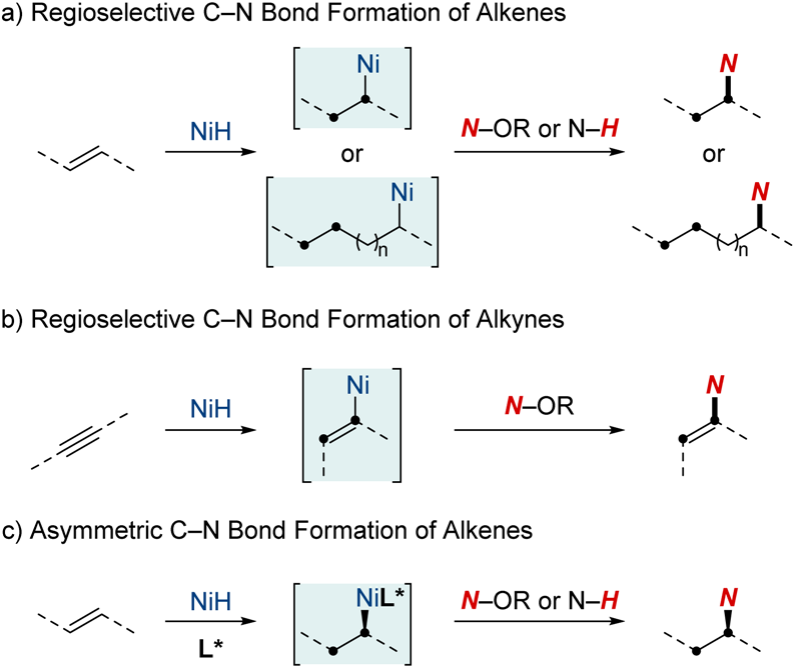
NiH-catalyzed hydroamination and hydroamidation of alkenes and alkynes.

This review will spotlight the unique attributes of nickel in the realm of metal hydrides, including techniques such as rapid chain-walking for remote functionalization and the utilization of coordination to achieve specific outcomes. While many individual studies have offered valuable insights, a review paper is pivotal for consolidating the wealth of knowledge from these developments. It aims to provide researchers and practitioners with a comprehensive perspective on contemporary techniques, mechanisms, and applications in C–N bond formation through NiH catalysis. This review is structured into three sections, each shedding light on distinct aspects of NiH-catalyzed hydroamination chemistry (Scheme 1). (1) Regioselective C–N bond formation of alkenes: in this section, we discuss the ways NiH catalysis facilitates the precise addition of amines to C–C double bonds. The discussion emphasizes the adaptability of this catalysis in handling diverse alkene substrates and its efficacy in optimizing the synthesis of vital nitrogen-based compounds. (2) Regioselective C–N bond formation of alkynes: turning our attention to alkynes, another class of unsaturated hydrocarbons, we elucidate how NiH catalysis plays a pivotal role in shaping alkynes, facilitating the synthesis of intricate molecules by the strategic integration of nitrogen atoms. (3) Asymmetric C–N bond formation: in this segment, we highlight the forefront of chiral molecule synthesis, showcasing advancements in NiH-catalyzed hydroamination. Specifically, we discuss the incorporation of asymmetric induction, paving the way for enantioselective transformations.

## 2. Regioselective NiH-catalyzed C–N bond formation of alkenes

### 2.1. NiH-catalyzed direct hydroamination/hydroamidation

At first glance, the direct hydroamination and hydroamidation of alkenes may appear to be straightforward processes. However, upon closer examination, the inherent challenges and complexities of these reactions become apparent, particularly in the context of regioselectivity. When it comes to alkene hydroamination and hydroamidation, achieving precise control over which carbon atom in the alkene bond reacts with the amine or amide is critical for the synthesis of the desired compounds. While this challenge is pertinent to all alkenes, it becomes particularly pronounced in the case of 1,2-disubstituted internal alkenes. The close electronic and steric similarities of the carbons in a 1,2-disubstituted alkene necessitate the development of innovative strategies. These strategies often entail the utilization of specific catalysts, ligands, or tailored reaction conditions designed to influence the regioselectivity of the reaction in the desired direction. Ongoing research remains essential to the development of more predictable and efficient methods for achieving regioselectivity, especially in the challenging context of 1,2-disubstituted alkenes.

**Scheme 2:**
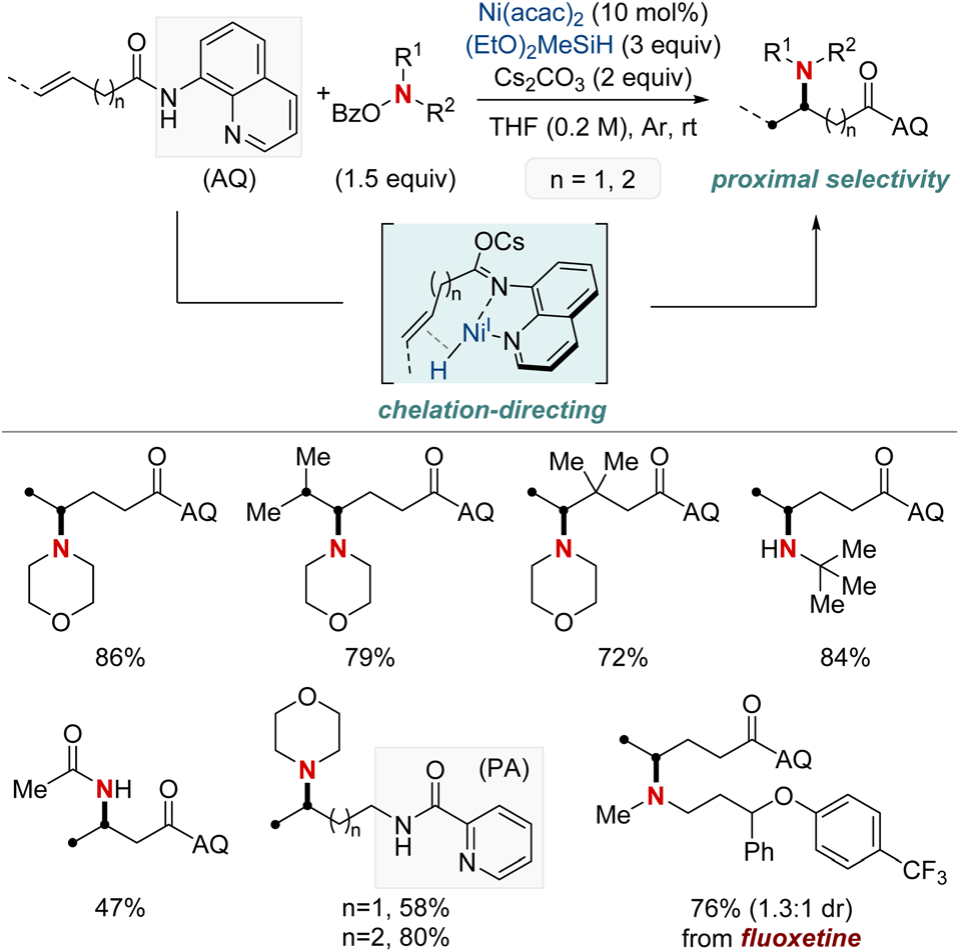
NiH-catalyzed proximal-selective hydroamination of unactivated alkenes.

In 2020, Hong *et al.* reported a strategy involving the NiH-catalyzed proximal-selective hydroamination of unactivated alkenes using aminobenzoates and hydrosilane (Scheme 2).^60^ This innovative methodology achieved regioselective NiH insertion into alkenes, with its efficacy attributed to the robust chelation between the bidentate directing group and nickel. Consequently, the nickel complex interacted with the aminating agent, leading to the formation of aminated derivatives. Notably, this method demonstrated remarkable versatility, accommodating a diverse array of primary and secondary amines. It also exhibited exceptional regiocontrol for both internal and terminal unactivated alkenes under mild conditions. By strategically employing aminoquinoline and picolinamide as bidentate directing groups, the research accomplished selective β-, γ-, and δ-aminations. This pivotal work, supported by both experimental and computational data, provided comprehensive insights into the reaction mechanism. It particularly highlighted the significance of migratory insertion steps as both the determinants of regioselectivity and turnover-limiting stages.

**Scheme 3:**
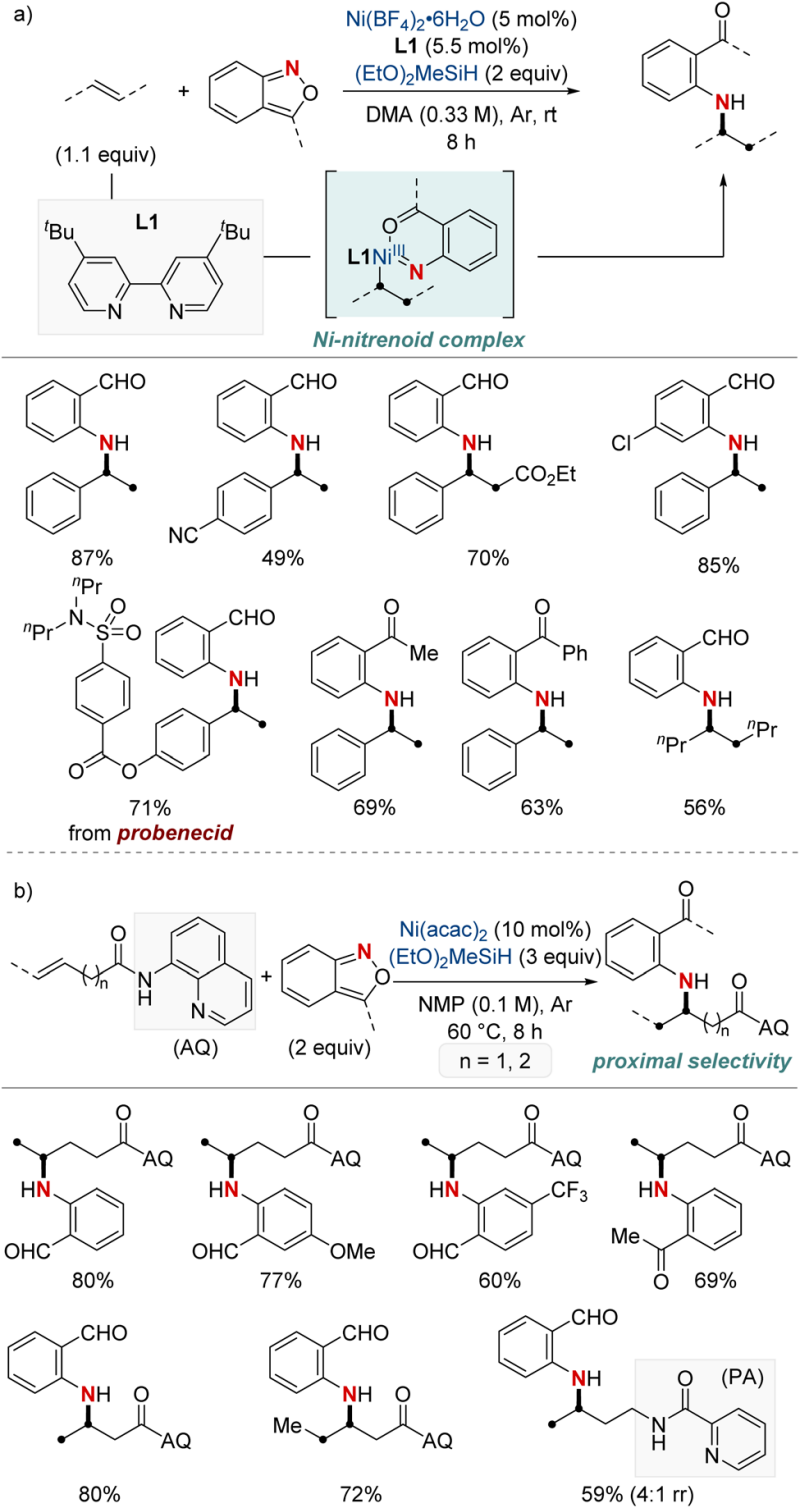
(a) NiH-catalyzed hydroarylamination. (b) NiH-catalyzed hydroarylamination of unactivated alkenes.

In 2021, Hu *et al.* reported a nickel-catalyzed hydroamination using anthranils as the electrophilic aminating agents (Scheme 3a).^61^ This method results in the synthesis of *N*-alkyl-2-aminobenzophenones, which serve as versatile intermediates. The process initiates with a migratory insertion during which a NiH complex, formed from the reaction of a bipyridine-type ligand-ligated nickel catalyst and hydrosilane, attaches at the benzylic position. Subsequently, this complex reacts with anthranils, giving rise to a nickel-nitrenoid complex. One distinctive aspect of this methodology is its unique reaction outcome compared to previous methods of copper hydride catalysis involving anthranils. While earlier processes led to a complete reduction resulting in benzyl alcohol, this method retains the carbonyl group, avoiding further reduction. Furthermore, the research emphasizes the adaptability of this method to different olefins and anthranils, highlighting its utility in producing a diverse array of valuable amination products. The protocol stands out for its excellent benzylic selectivity, mild reaction conditions, and broad substrate range. It also proves highly effective in the late-stage modification of numerous pharmaceutical derivatives.

Subsequently, this research group developed a NiH-catalyzed hydroamination with proximal selectivity for unactivated alkenes bearing bidentate directing groups, employing anthranils as amination agents (Scheme 3b).^62^ This method builds upon the prior work of the Hong group in 2020, which also leveraged bidentate directing groups, highlighting the versatility of regioselective hydroamination techniques. The method effectively enables the incorporation of a wide range of primary arylamines with an *ortho*-carbonyl group into both terminal and internal unactivated alkenes. This results in the synthesis of invaluable β- and γ-amino acid precursors, characterized by remarkable regiocontrol. Furthermore, the effectiveness and broad applicability of this method are evident in its capacity to convert multifunctionalized arylamines into *N*-heterocycles.

**Scheme 4:**
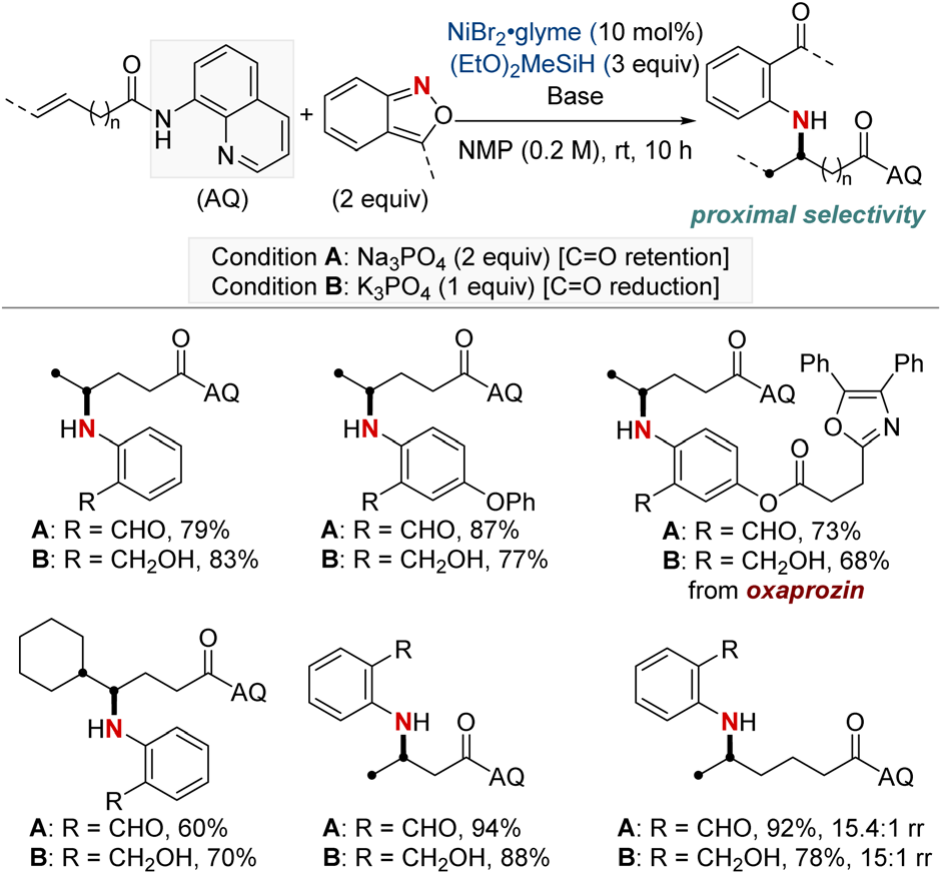
NiH-catalyzed proximal-selective divergent hydroaryl-amination.

In 2023, Wang and Zheng *et al.* presented a method closely resembling the one described in the previous approach by Hu and Huo, which focuses on the hydroamination of unactivated alkenes using anthranils (Scheme 4).^63^ This approach distinguishes itself by categorizing the reaction conditions into two specific modes: one that preserves the carbonyl group and another that leads to alcohol formation through complete reduction, depending on the choice of bases. This method offered selective synthesis of a wide range of arylamino aldehydes and alcohols. In addition, it expands upon the prior work by examining alkenes in which the double bond is further distant from the carbonyl group. The authors observed favorable reactivity and appreciable regioselectivity for β,γ- and γ,δ-alkenes, as well as δ,ε-alkenes. However, ε,ζ-alkenes demonstrated only moderate reactivity with a near 1 : 1 regioselectivity. This method offers a useful framework for controlling the reaction outcome, allowing for the retention of the carbonyl group or the production of alcohols based on specific conditions and preferences.

**Scheme 5:**
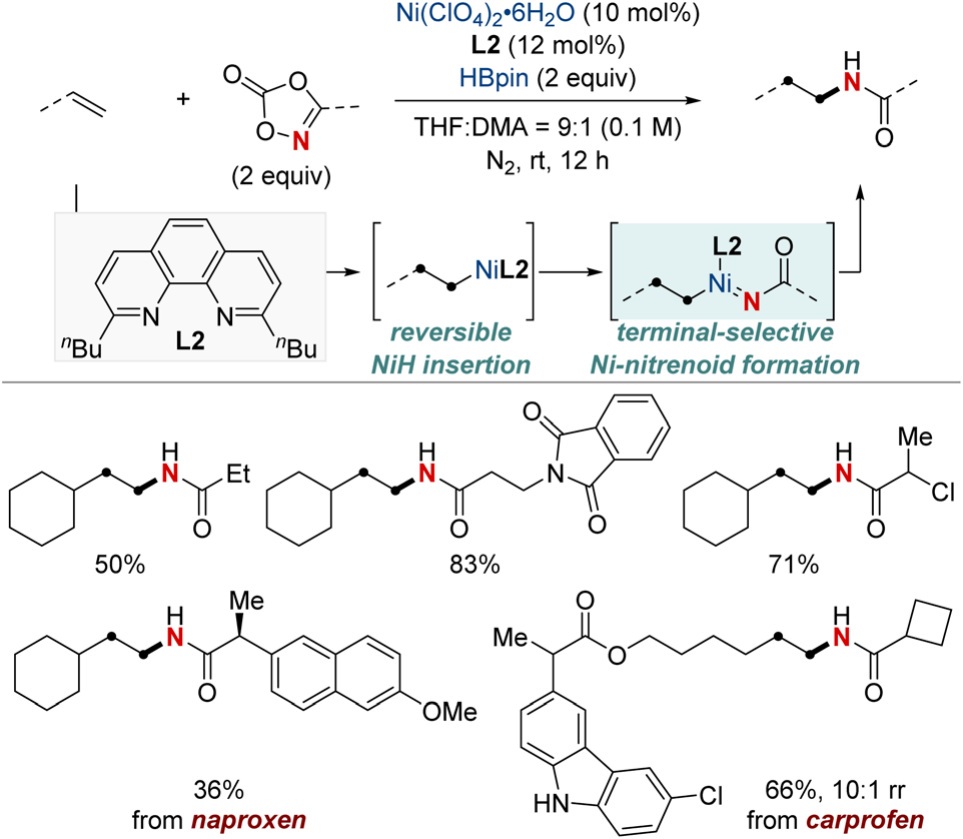
NiH-catalyzed *anti*-Markovnikov hydroamidation of unactivated terminal alkenes.

In 2022, Yu and Lin *et al.* reported a NiH-catalyzed intermolecular hydroamidation using dioxazolones and pinacol borane that exhibits *anti*-Markovnikov selectivity (Scheme 5).^64^ This method employs 2,9-dibutylphenathroline (diBuphen) as a ligand, and the steric bulk of this ligand plays a pivotal role in facilitating the selective incorporation of nickel primarily at the terminal position. Subsequently, the nickel-alkyl intermediate reacts with dioxazolones to form a nickel-nitrenoid, ultimately leading to the formation of amidated products through C–N bond formation for the synthesis of various *N*-alkyl amides. A standout feature of this method is its versatility; it accommodates a diverse range of dioxazolones and is suitable for both terminal and internal alkenes, encompassing even natural products with multiple functional groups. Mechanistic insights, enriched through deuterium labeling studies and trapping reagent experiments, shed light on a reversible insertion/elimination process of the NiH into the alkene, followed by an irreversible amidation step. Additional calculations suggest the involvement of the Curtin–Hammett selectivity model in this process.

### 2.2. NiH-catalyzed migratory hydroamination/hydroamidation

NiH-catalyzed migratory hydrofunctionalization is a robust synthetic technique that selectively modifies aliphatic C(sp^3^)–H bonds.^65^ This process unveils new horizons for transforming molecules at positions traditionally considered challenging to access. The intricacies of reaction conditions, ligand characteristics, and substrate specificities frequently require meticulous optimization. Despite the challenges, the undeniable potential of migratory hydroamination and hydroamidation places them at the forefront of contemporary synthetic research. In the following section, our focus is on recent advances in this field using NiH catalysis.

**Scheme 6:**
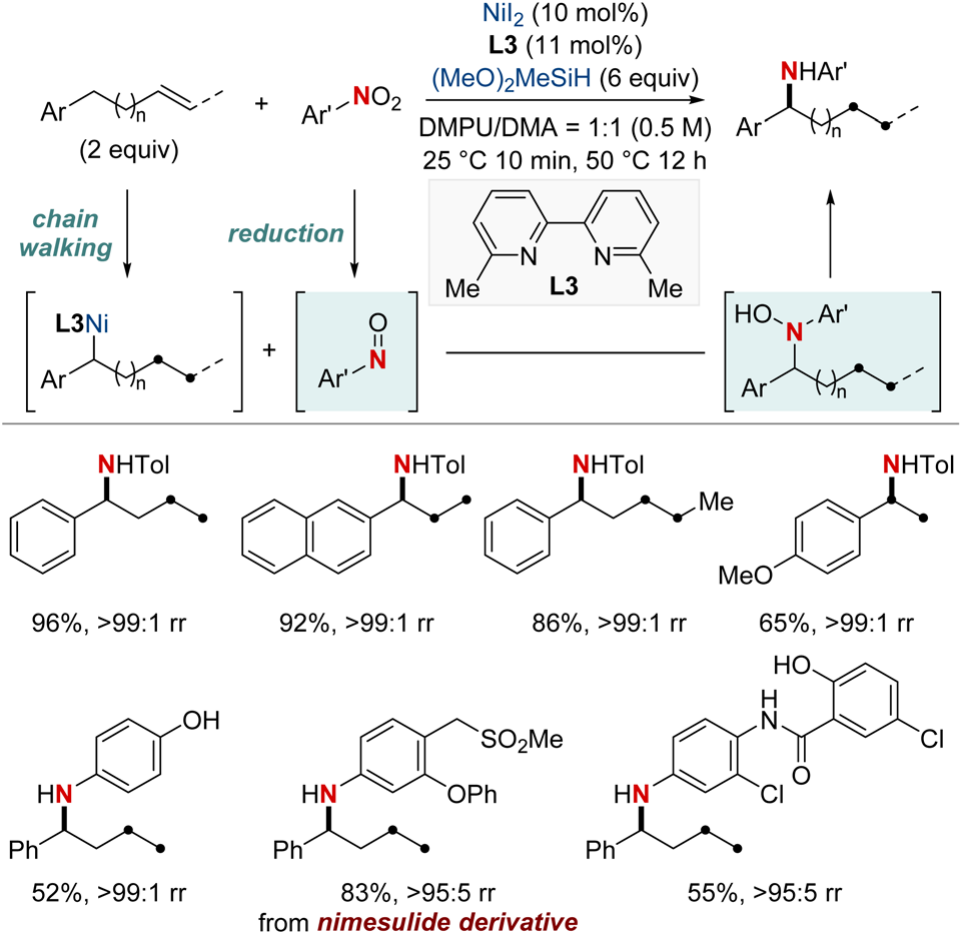
NiH-catalyzed benzylic-selective migratory hydroarylamination using nitroarenes.

In 2018, Zhu *et al.* developed a NiH-catalyzed remote relay hydroarylamination process (Scheme 6).^66^ This innovative approach allowed the introduction of arylamino groups at benzylic positions within alkyl chains, harnessing an intricate chain-walking mechanism. During the process, once the NiH was integrated into the alkene, it embarked on a meticulous chain-walking process. This involved a series of successive β-hydride elimination and migratory insertion steps, eventually culminating at the benzylic position, where it forged a thermodynamically stable alkyl–nickel complex. This resultant complex was then primed to engage in the hydroarylamination reaction. It paired with the nitrosoarene, an intermediate derived from the efficient reduction of nitroarene by the NiH. This protocol provided an efficient route for the synthesis of valuable arylamines, directly from basic olefins and nitro(hetero)arenes. The versatility of this method was highlighted by its ability to achieve regioconvergent arylamination of isomeric olefin mixtures.

**Scheme 7:**
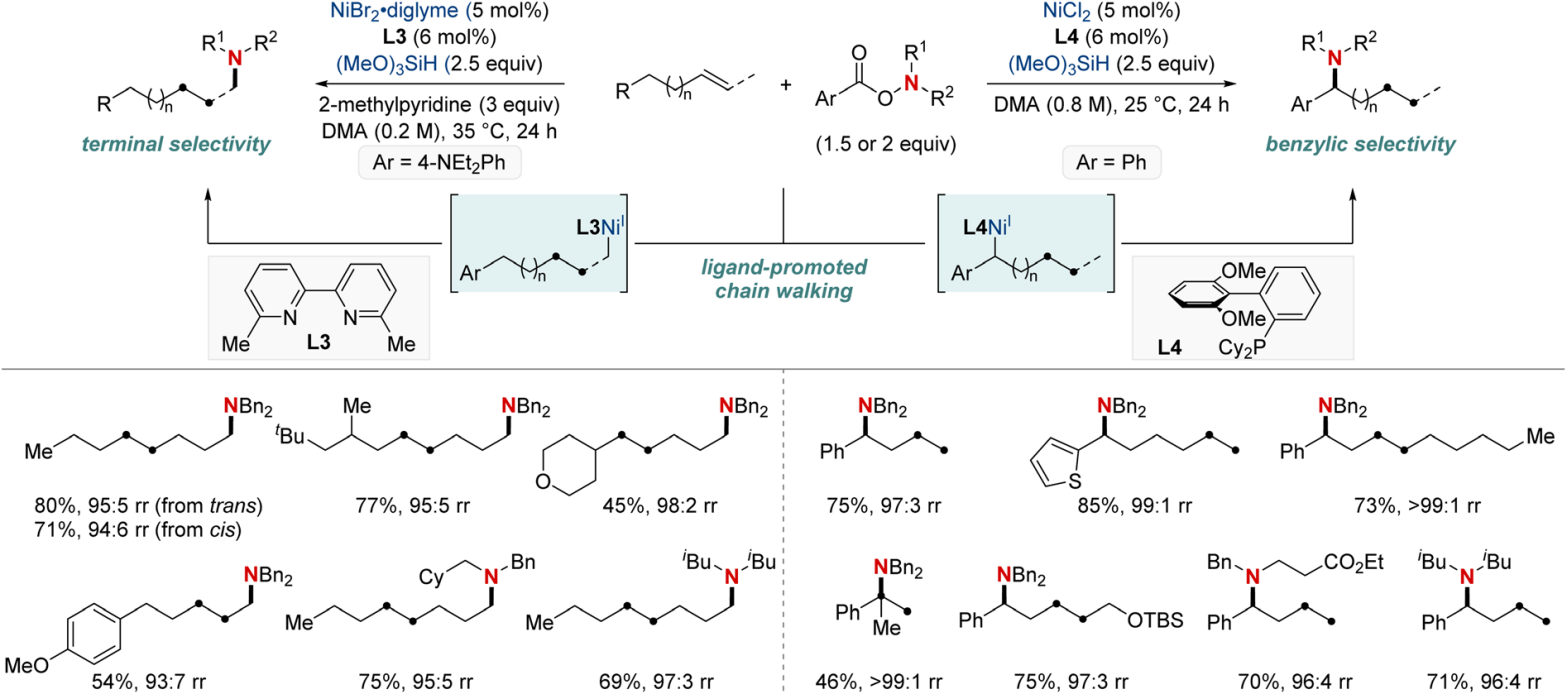
NiH-catalyzed terminal- and benzylic selective migratory hydroamination using ligand effects.

In 2020, building on their previous studies, the same group further advanced their methodology by unveiling a NiH-catalyzed migratory hydroamination procedure, employing aminobenzoate electrophiles (Scheme 7).^67^ This refined method facilitated the regiodivergent placement of a distal amino group at either the benzylic or terminal locations within alkyl chains. The choice between monophosphine-type ligands and bipyridine-type ligands determined the specific positioning. Impressively, this approach is both practical and efficient making use of easily accessible olefins. Furthermore, the group showcased that an alkyl bromide could be converted into an olefin when combined with Mn(0) as a reducing agent. The process exhibited remarkable compatibility with substrates containing a wide range of functional groups and achieved remote C(sp^3^)–H amination products with impressive yields and regioselectivity, maintaining these attributes even when scaled up to 10 mmol. Importantly, this technique was adeptly applied to the regioconvergent transformation of petroleum-based feedstocks. The efficient transformation of isomeric olefin mixtures into a single, valuable amine compound variant further highlighted its potential.

**Scheme 8:**
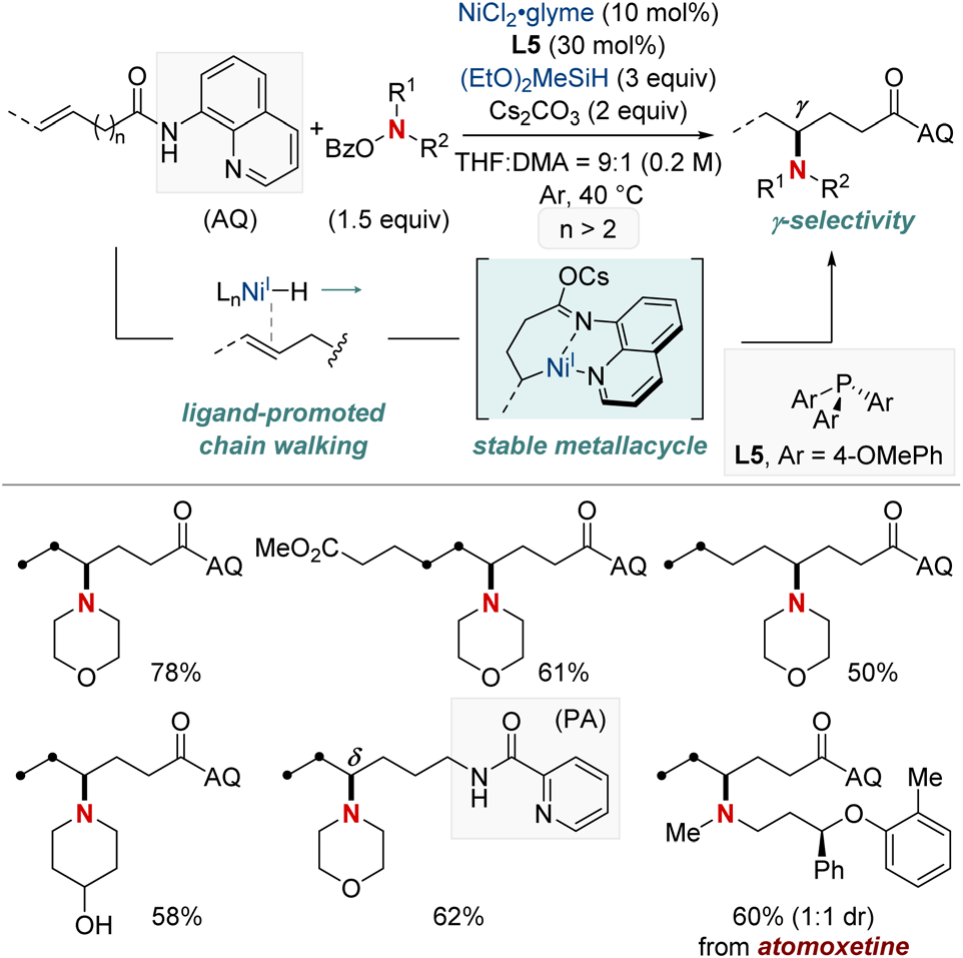
NiH-catalyzed γ-C(sp^3^)–H-selective migratory hydro-amination of unactivated alkenes.

In 2021, Hong *et al.* unveiled a groundbreaking NiH-catalyzed γ-selective migratory hydroamination of alkenyl amides, leveraging both a bidentate directing group and phosphine ligand (Scheme 8).^68^ This innovative strategy represents the first instance of γ-C(sp^3^)–H functionalization *via* a chain-walking approach, distinguishing itself from previous methods that were confined to α- and β-C(sp^3^)–H functionalizations. The process is facilitated by phosphine ligands, culminating in the formation of a 6-membered nickellacycle. This sequence is guided by an 8-aminoquinoline directing group and is eventually intercepted by an aminobenzoate. This method allows for the introduction of a diverse array of amines at the γ-C(sp^3^)–H bond of unactivated alkenes, irrespective of their alkyl chain lengths, paving the way for the streamlined synthesis of valuable γ-aminated derivatives. Moreover, the researchers extended the technique to δ-selective amination, underscoring its adaptability with picolinamide-integrated alkene substrates. The intricate chain-walking mechanism and its selective pathways were elucidated using deuterium experiments and rigorous computational analysis.

**Scheme 9:**
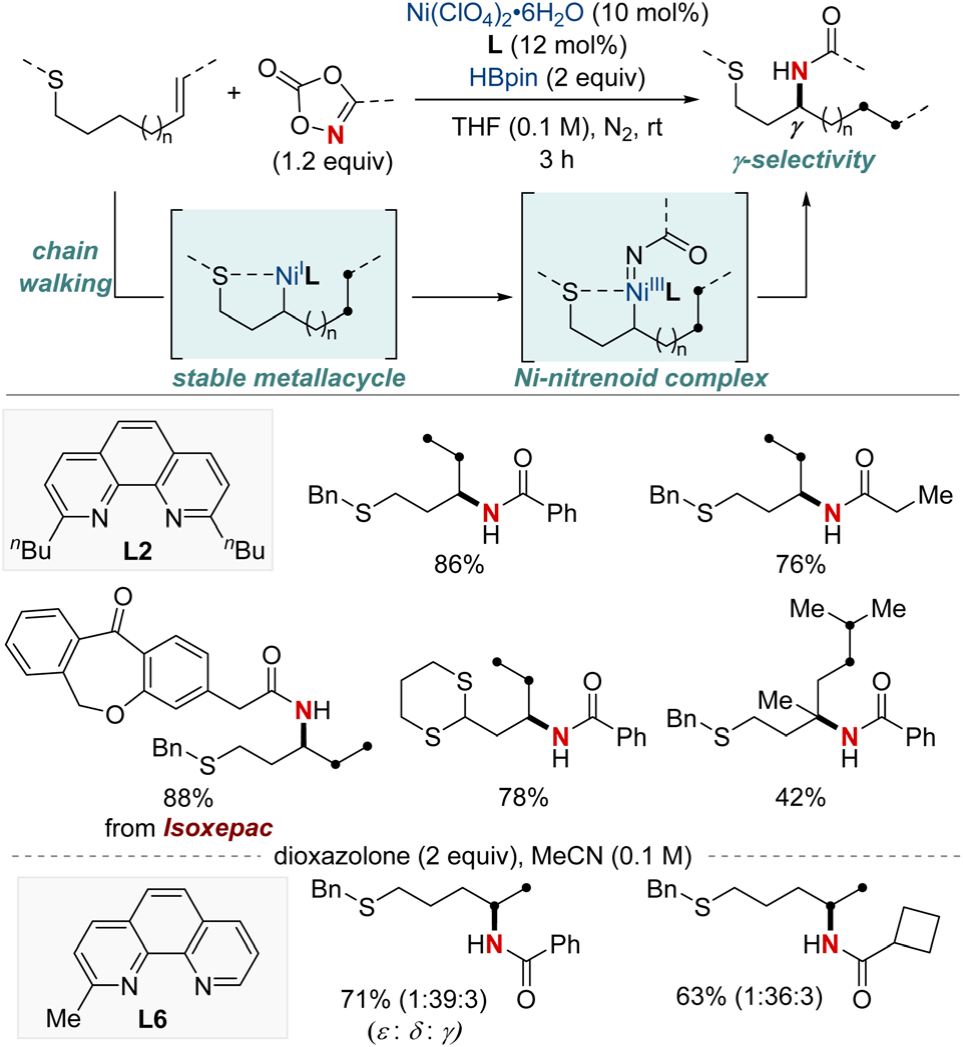
NiH-catalyzed γ-C(sp^3^)–H-selective migratory hydro-amidation of unactivated alkenes.

Yu and colleagues reported a NiH-catalyzed thioether-directed cyclometalation strategy, enabling γ-selective migratory hydroamidation of thioether-containing unactivated alkenes (Scheme 9).^69^ This innovative approach utilizes a phenanthroline derivative as a ligand and dioxazolones as amidating reagents to selectively target γ-C(sp^3^)–H bonds, yielding amide products with remarkable regioselectivity and high yields. The preference for five-membered nickelacycle formation effectively curtails chain-walking isomerization at a specific γ-methylene site. Such selectivity ensures distinct regiodifferentiation amidst similar chemical environments present within the hydrocarbon sequence. Furthermore, this protocol demonstrated that a ligand bearing a single methyl group furnished the Markovnikov product for the terminal δ,ε-alkene substrates. Interestingly, the reaction proceeds even when the substrate–nickel complex, which features a sulfide bond to the nickel center, is isolated and exposed to the standard reaction conditions. This catalytic hydroamidation protocol not only serves as a versatile tool for diverse amide synthesis but also provides insights into the development of regiocontrolled C–N bond formation in unactivated aliphatic alkanes.

**Scheme 10:**
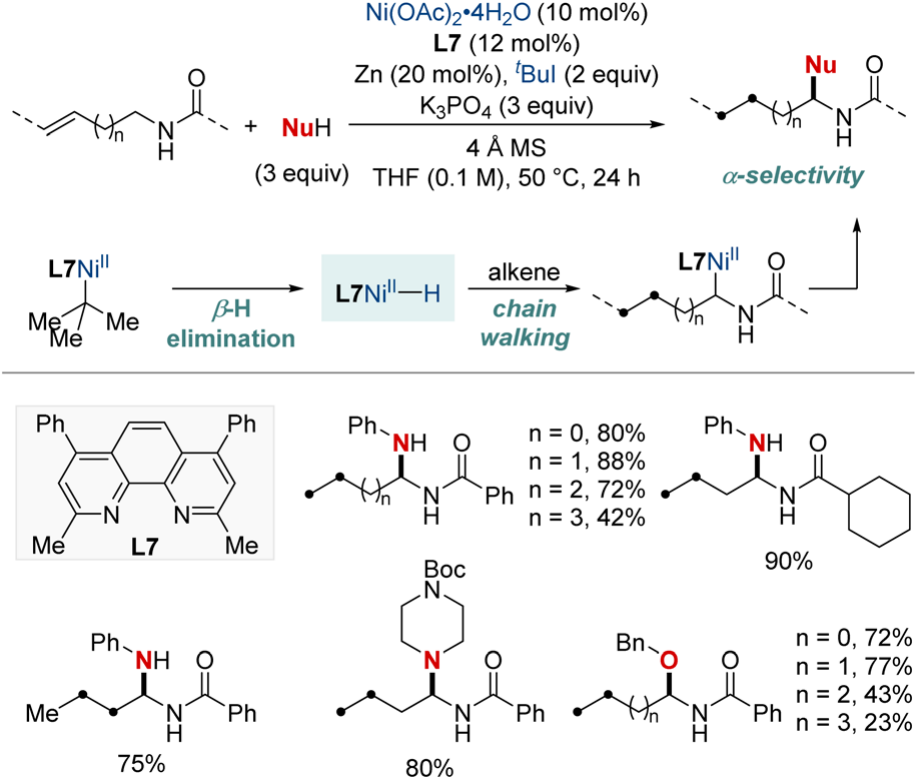
NiH-catalyzed migratory hydroamination using free N–H amine.

In 2023, Zhang and Yuan *et al.* introduced an efficient nickel-catalyzed method for remote hydroamination and hydroetherification of alkenes using amines and alcohols as coupling nucleophiles (Scheme 10).^70^ This method proficiently synthesized gem-diamine and *N*,*O*-acetal derivatives, achieving commendable yields and exhibiting exclusive regioselectivity with the assistance of a phenanthroline-type ligand. This breakthrough overcomes previous challenges of modifying alkenes with traditionally unreactive amines and alcohols. The key to the success of this method was the incorporation of 2-iodo-2-methylpropane (^*t*^BuI), which functioned both as a hydride donor and a radical precursor. This study presents an innovative approach for the incorporation of diverse amino or alkoxyl functionalities onto C(sp^3^)–H sites distant from alkene's double bond. The methodology demonstrated its robustness with a broad substrate scope and the regioconvergent transformation of mixed alkenes into a single remote functionalized product. Additionally, DFT computational analyses shed light on the underlying mechanistic route, emphasizing the chain-walking process that ultimately results in a thermodynamically favorable five-membered nickellacycle.

## 3. NiH-catalyzed regioselective C–N bond formation of alkynes

The hydroamination of alkynes has become a cornerstone in synthetic chemistry, allowing the introduction of an amine group across a carbon–carbon triple bond to produce enamines or imines. These intermediates are pivotal in organic synthesis because of their potential to be transformed into various functionalized compounds. Historically, achieving selective hydroamination of alkynes was challenging, especially under mild conditions with diverse substrates. However, NiH-catalyzed reactions stand out for their excellent regiocontrol in mild conditions. This method adeptly creates nitrogen-rich structures often found in pharmaceuticals, agrochemicals, and natural products. Ongoing research hints at more refined methods, broader substrate applicability, and even more advanced and sustainable catalytic systems.

**Scheme 11:**
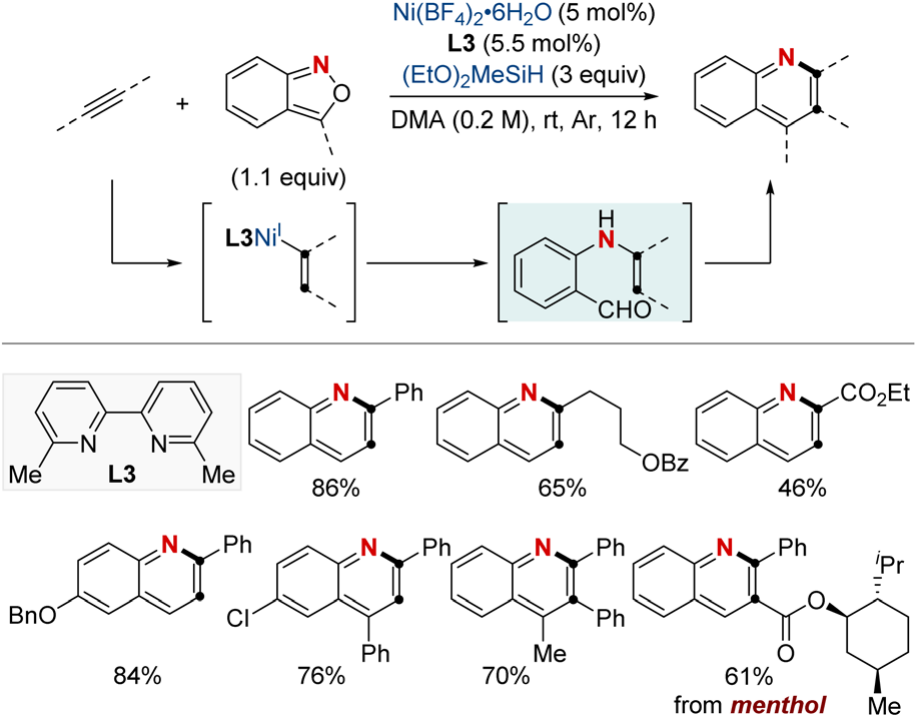
NiH-catalyzed quinoline synthesis using alkynes and anthranils.

In 2021, the Hu group unveiled an efficient NiH-catalyzed hydroamination/cyclization cascade of alkynes and anthranils, offering a convenient route for synthesizing elaborately substituted quinolines (Scheme 11).^71^ This method stood out for its high regioselectivity, mild operating conditions, and extensive substrate compatibility, embracing a variety of alkynes ranging from terminal to internal and from aryl to alkyl, inclusive of both electron-deficient and electron-rich types. Notably, the bipyridine-ligated NiH showed a Markovnikov-type migratory insertion with terminal alkynes, while favoring the aryl group with internal ones. The versatility of this approach also enabled the late-stage functionalization of natural products and the synthesis of crucial compounds, including the antitumor agent graveolinine and a potent triplex DNA intercalator. Such advancements in NiH catalysis hint at a promising future for transforming basic alkynes into a diverse array of high-value compounds.

**Scheme 12:**
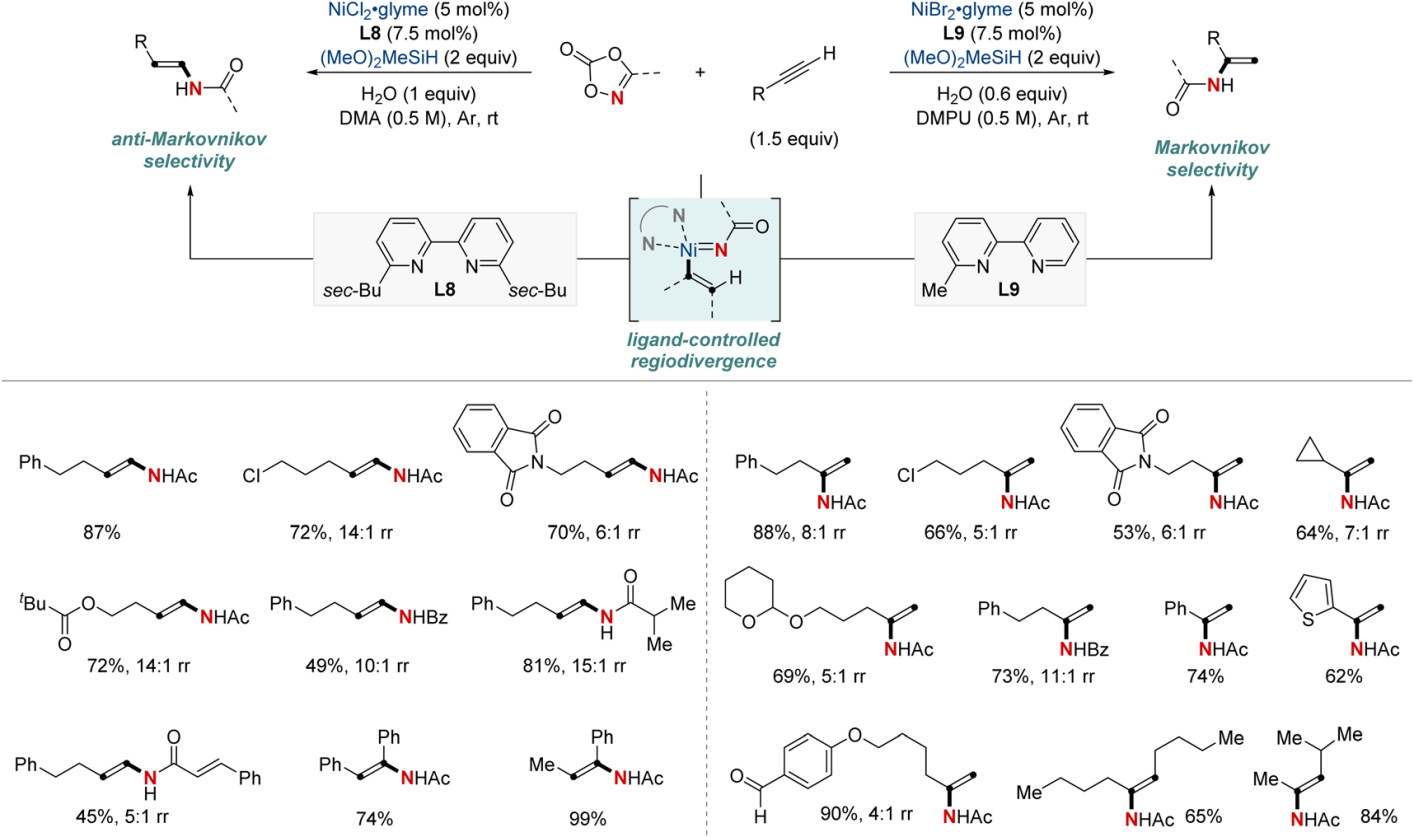
NiH-catalyzed regiodivergent hydroamidation of alkynes.

In 2021, Chang, Seo, and colleagues introduced a groundbreaking NiH-catalyzed strategy for the formal hydroamidation of alkynes using dioxazolones, facilitating the synthesis of valuable secondary enamides. Selectivity between (*E*)-*anti*-Markovnikov or Markovnikov was determined by the choice of bipyridine ligands, being either disubstituted or monosubstituted (Scheme 12).^72^ This approach is versatile, accommodating both terminal and internal alkynes, and it exhibits tolerance toward various functional groups. Significantly, it effectively overcame challenges related to semireduction pathways by employing a low-energy inner-sphere nitrenoid transfer. The method consistently achieved good to excellent regioselectivity, and the introduction of H_2_O proved essential for attaining high catalyst turnovers by facilitating transmetalation. This versatility extended to the construction of synthetically valuable alkylamide moieties, including enantioselective transformations through sequential asymmetric hydrogenation, highlighting its wide range of synthetic applications. Mechanistically, this work underscores a unique Ni-nitrenoid intermediacy, pointing to future C–N bond-forming methodologies.

**Scheme 13:**
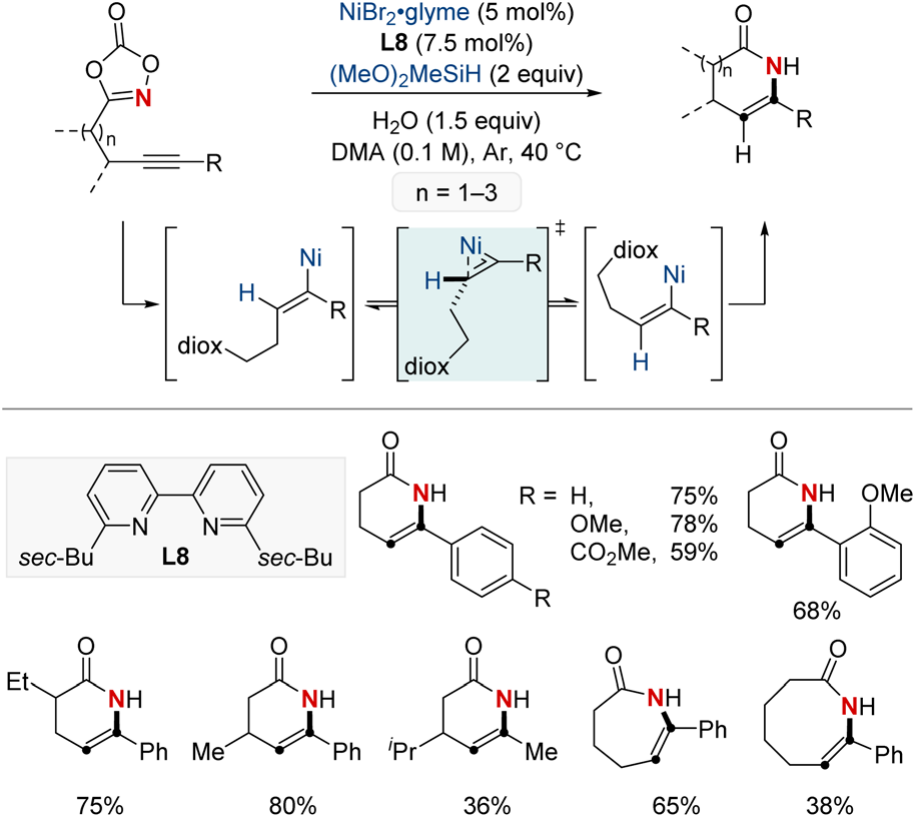
NiH-catalyzed *endo*-selective intramolecular hydroamidation of alkynes.

In 2022, the same group introduced a NiH-catalyzed intramolecular hydroamidation of alkynyl dioxazolones, achieving precise endo-selective C–N bond formation, which produces various six-to eight-membered endocyclic enamides from a wide range of alkynes (Scheme 13).^73^ This novel process harnesses Ni(i) catalysis and progresses through a series of regioselective steps: *syn*-hydronickelation, alkenylnickel *E*/*Z* isomerization, oxidative activation of dioxazolone, and inner-sphere nitrenoid transfer. Mechanistic insights disclosed the η^2^-vinyl-like transition state governing the key alkenylnickel isomerization, a previously unclarified step. Synthetically, this method shines by allowing the diastereoselective creation of rich δ-lactams, emphasizing the enamide's adaptability for varied transformations. This research not only pioneers in endo-selective cyclizations but also sets the stage for NiH-catalyzed C–N bond formation, enriching the resources for crafting valuable cyclic enamides.

## 4. Advancements in asymmetric hydroamination and hydroamidation of alkenes

### 4.1. Asymmetric hydroamination/hydroamidation at activated sites

The catalytic formation of enantiopure aliphatic amines and amides from abundant and readily accessible starting materials has persistently stood as a formidable challenge in synthetic chemistry. The success of such transformations largely hinges on the design of chiral ligands. In this section, we explore the latest advancements in asymmetric hydroamination and hydroamidation of activated systems.

**Scheme 14:**
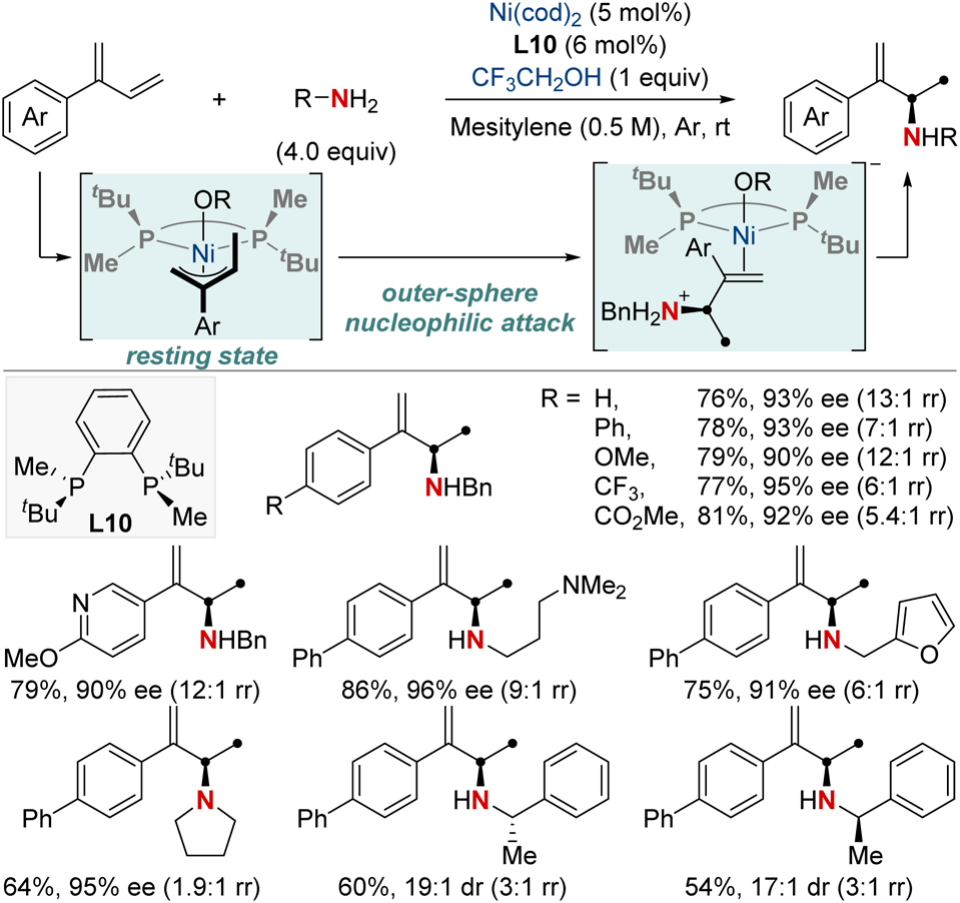
NiH-catalyzed asymmetric 1,2-hydroamination of branched dienes.

In 2019, the Mazet group unveiled a Ni-catalyzed enantioselective hydroamination of branched 1,3-dienes, marking a significant milestone in synthetic chemistry (Scheme 14).^74^ This method stands out due to its unparalleled regio-, chemo-, and enantioselectivity, providing an efficient avenue to produce high-value chiral allylic amines using both primary and secondary amines. In addition, this approach eliminates the dependency on often complex amination reagents. To gain deeper insights into the intricate mechanisms, the group undertook comprehensive studies, including NMR tracking of the reaction, isotopic labeling using deuterium (^2^H) to map reaction routes, and kinetic assessments to identify the crucial rate-determining step. Their findings highlighted the catalytic resting state as a Ni−π-allyl complex and identified the pivotal step as an outer-sphere nucleophilic attack, made possible by H-bonded amine groupings, commonly seen as dimers. These insights not only optimized reaction conditions but also broadened the method's applicability to various functional groups. This research showcased a streamlined approach for the preparation of enantioenriched chiral primary allylamines.

**Scheme 15:**
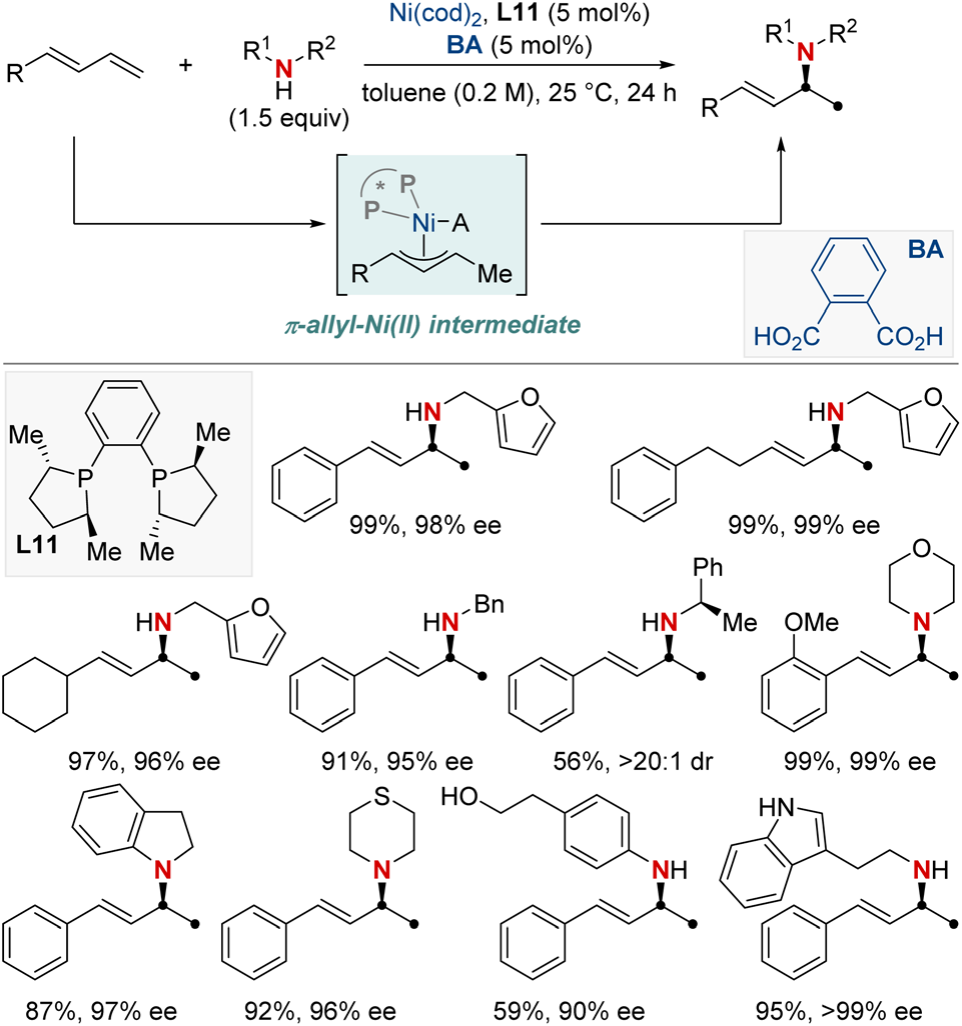
NiH-catalyzed asymmetric 1,2-hydroamination of linear dienes.

In the same year, Yin and colleagues unveiled an innovative nickel/Brønsted acid-catalyzed asymmetric hydroamination reaction (Scheme 15).^75^ This method efficiently converts a broad spectrum of primary and secondary amines into highly enantioenriched allylic amines under mild conditions. Notably, it exhibits exceptional chemoselectivity for amines possessing multiple nucleophilic sites. Its success hinges on the pairing of chiral bisphosphine ligands with the right Brønsted acids, broadening the scope of accessible enantioenriched secondary and tertiary allylic amines and enabling late-stage modifications of complex molecules. The method also demonstrates compatibility with a diverse array of functional groups and heterocycles. Mechanistic investigations showed the carbon–nitrogen bond formation step to be reversible. Notably, racemization is observed in reactions using secondary amines over prolonged periods, while primary amines remain stable. This makes the approach exceptionally promising for yielding enantioenriched amines, holding significant implications for medicinal chemistry and innovative reaction methodologies.

**Scheme 16:**
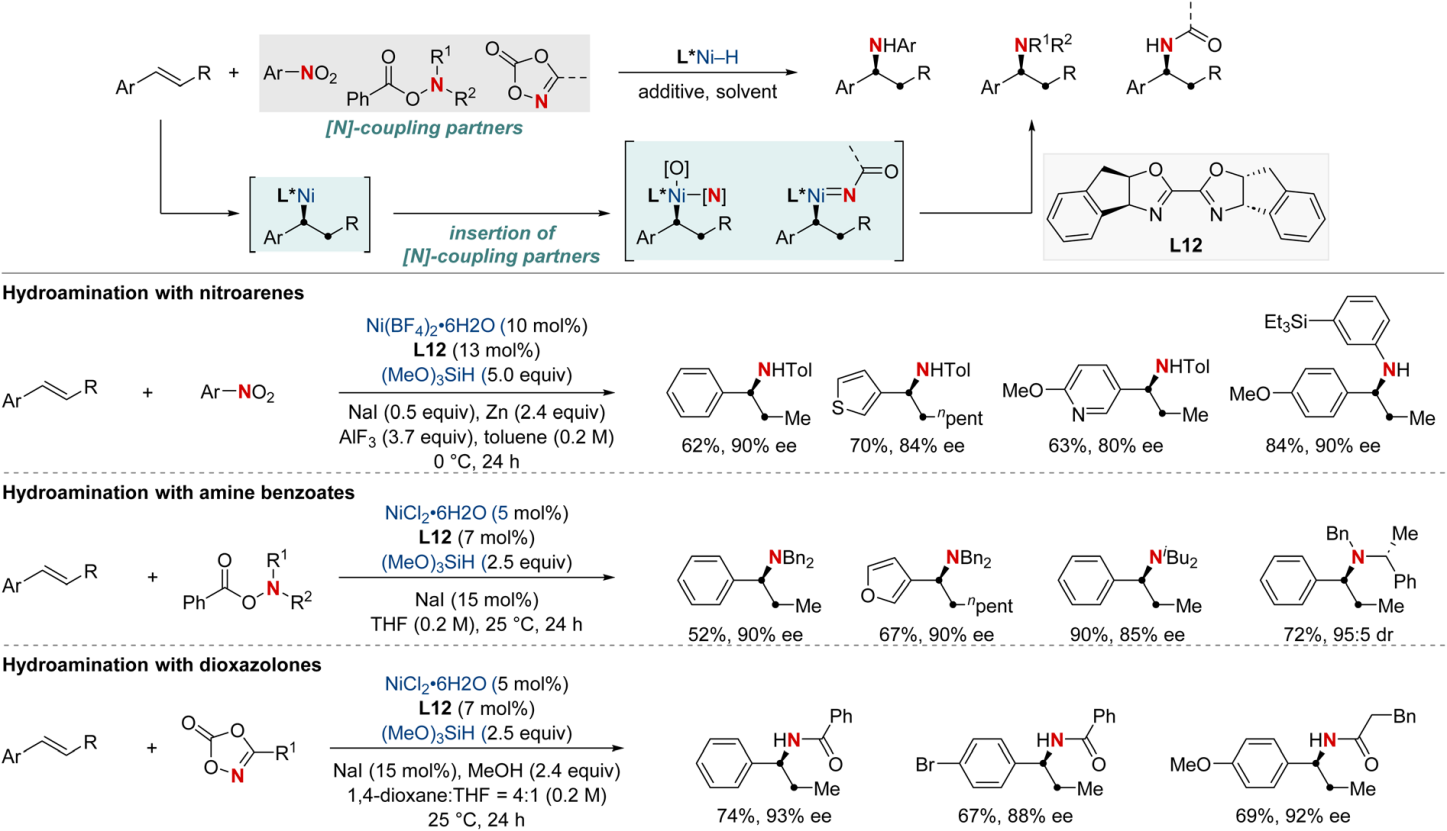
NiH-catalyzed asymmetric hydroarylamination, hydroamination, and hydroamidation of vinylarenes.

In 2021, the Zhu group advanced asymmetric catalysis by introducing a pioneering platform for regio- and enantioselective hydroarylamination, hydroalkylamination, and hydroamidation of styrenes (Scheme 16).^76^ Central to this breakthrough is the use of NiH catalysis combined with a straightforward bioxazoline ligand, functioning under mild conditions. Beyond furnishing enantioenriched benzylic arylamines, alkylamines, and amides, the methodology further demonstrates its adaptability by incorporating nitroarenes, hydroxylamines, and dioxazolones as versatile amination and amidation agents. Mechanistic insights suggest that chiral induction in these reactions stems from an enantiodifferentiating *syn*-hydronickellation step, adding further depth to this transformative approach. This cutting-edge approach holds immense promise for synthesizing enantioenriched compounds and offers potential applications across various domains of synthetic chemistry.

**Scheme 17:**
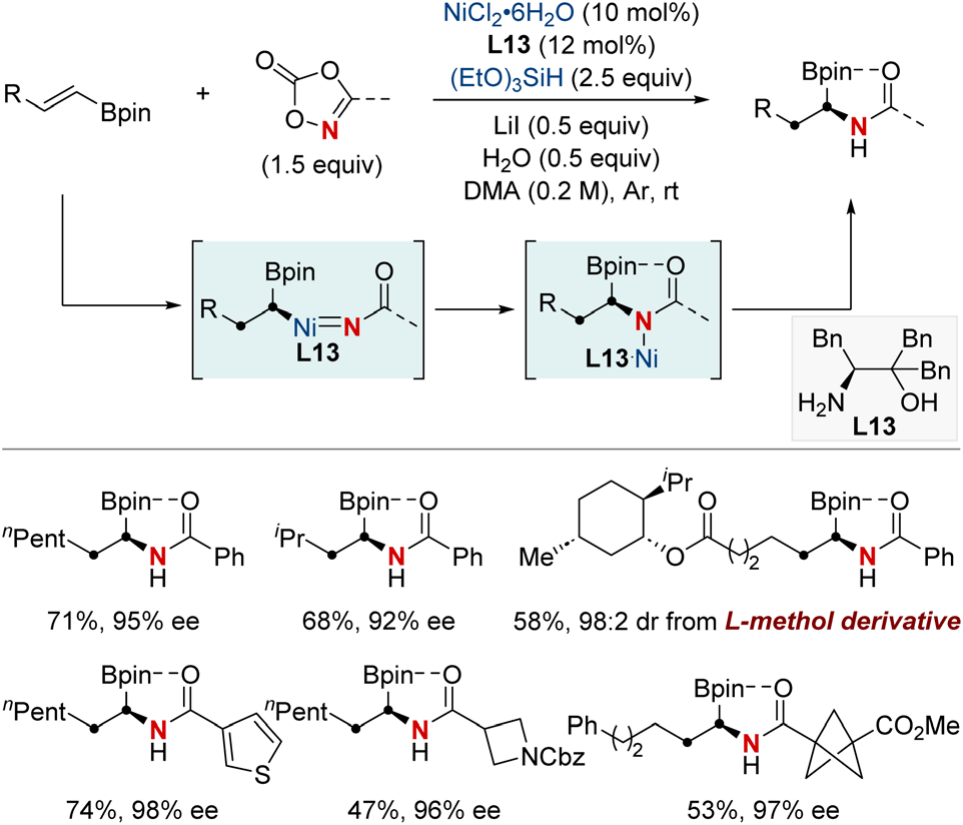
NiH-catalyzed asymmetric hydroamidation of vinyl boronates.

In 2022, the Zhu group achieved a significant advancement in the field of chiral α-aminoboronic acids and their derivatives, recognized for their role as bioactive compounds and approved therapeutic agents (Scheme 17).^77^ This study unveiled a NiH-catalyzed asymmetric hydroamidation process, facilitated by a straightforward and effective amino alcohol ligand. This method proficiently produces a broad range of enantioenriched α-aminoboronates under benign conditions. The suggested reaction pathway commences with an enantioselective hydrometallation, evolving into a finely-tuned inner-sphere nitrenoid transfer, leading to C–N bond generation. The synthetic utility was further exemplified by a three-step synthesis of vaborbactam, a β-lactamase inhibitor, thereby streamlining the original six-step synthetic approach. This breakthrough has the potential to significantly impact the production of chiral aminoboronic acids, with implications for the pharmaceutical industry.

**Scheme 18:**
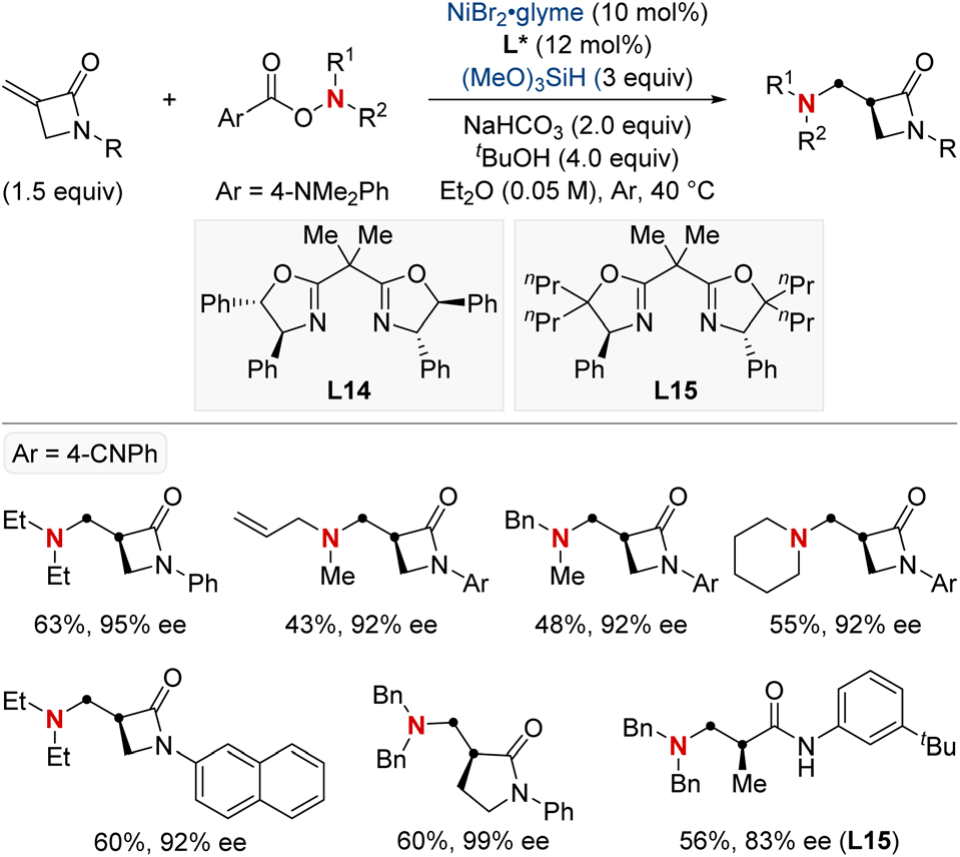
NiH-catalyzed asymmetric hydroamination of α,β-unsaturated amides.

In 2023, the Shu group embarked on a landmark project in synthetic chemistry, driven by the critical role of enantioenriched α-chiral-β-amino acid derivatives in both biological and pharmaceutical fields. This research led to the development of a unique enantioselective formal hydroamination method (Scheme 18).^78^ This method was specifically tailored for *N*,*N*-disubstituted acrylamides, making the synthesis of enantioenriched α-chiral-β-aminoamide derivatives more efficient. Impressively, this technique overcame challenges typically encountered with electronically disfavored hydroamination reactions. This success was achieved by harnessing a NiH-catalyzed *anti*-Markovnikov-selective formal hydroamination of alkenes, paving the way for producing the desired α-chiral-β-aminoamide derivatives. Showcasing impressive adaptability to a broad spectrum of functional groups, the procedure reliably yielded a diverse set of α-chiral-β-aminoamide derivatives. Furthermore, these compounds were crafted with exceptional efficiency and unparalleled enantioselectivity, underscoring the method's transformative potential in synthetic chemistry.

**Scheme 19:**
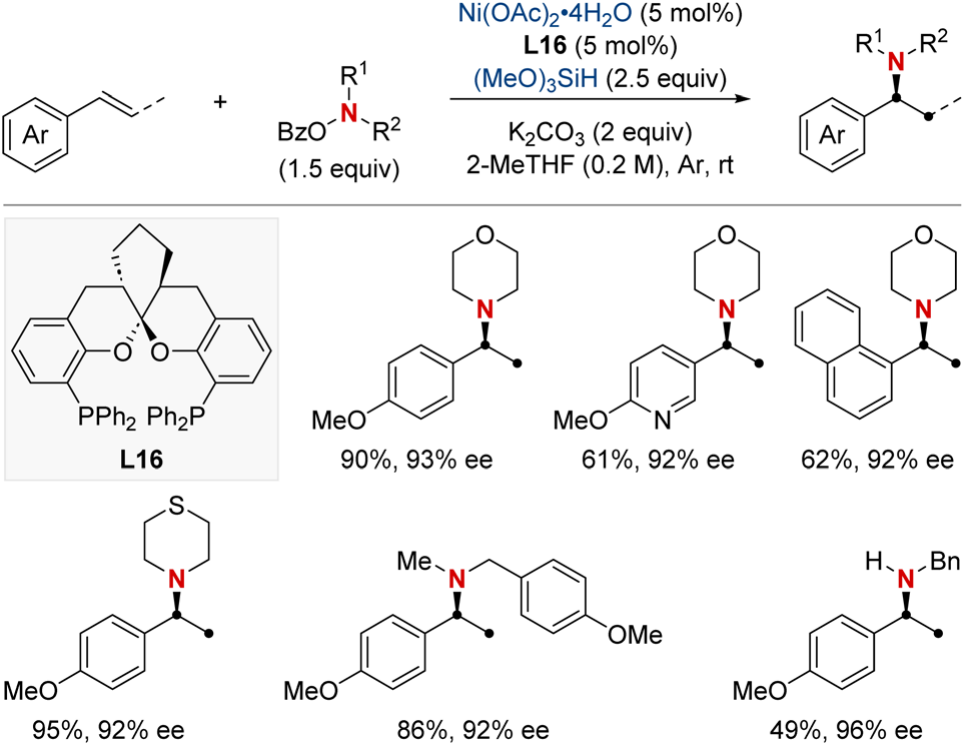
NiH-catalyzed asymmetric hydroamination of vinyl arenes.

In that same year, Ding and his colleagues made significant breakthroughs by unveiling a Ni-catalyzed enantioselective hydroamination procedure tailored for vinylarenes (Scheme 19).^79^ This approach was instrumental in the streamlined synthesis of a vast array of α-branched chiral alkylamines. The method's standout feature was its unique Markovnikov regioselectivity paired with unparalleled enantioselectivity. Central to this technique's success was the SKP (spiroketal phosphine) ligand, which played a pivotal role. Its incorporation significantly boosted the reaction's reactivity and fine-tuned the enantiocontrol. Illustrating the broad versatility of their approach, the group successfully executed gram-scale reactions and showcased the technique's prowess in the precise modification of molecules with medicinal relevance. In pursuit of a deeper understanding of the process, deuterium-labeling experiments were undertaken, suggesting the irreversible hydronickelation of vinylarenes as the likely foundation for achieving such high enantioselectivity.

### 4.2. Asymmetric hydroamination/hydroamidation at unactivated sites

In this section, we explore the forefront of progress in asymmetric hydroamination and hydroamidation involving previously challenging unactivated alkenes. Central to our discussion is the enduring quest to synthesize enantiopure aliphatic amines and amides, utilizing commonly encountered starting materials. The intricate design and deployment of chiral ligands stand out as crucial elements, guiding the quest to realize this esteemed objective in synthetic chemistry. We cover a wide spectrum of both intermolecular and intramolecular reactions, highlighting the vast versatility and untapped potential of these contemporary approaches.

**Scheme 20:**
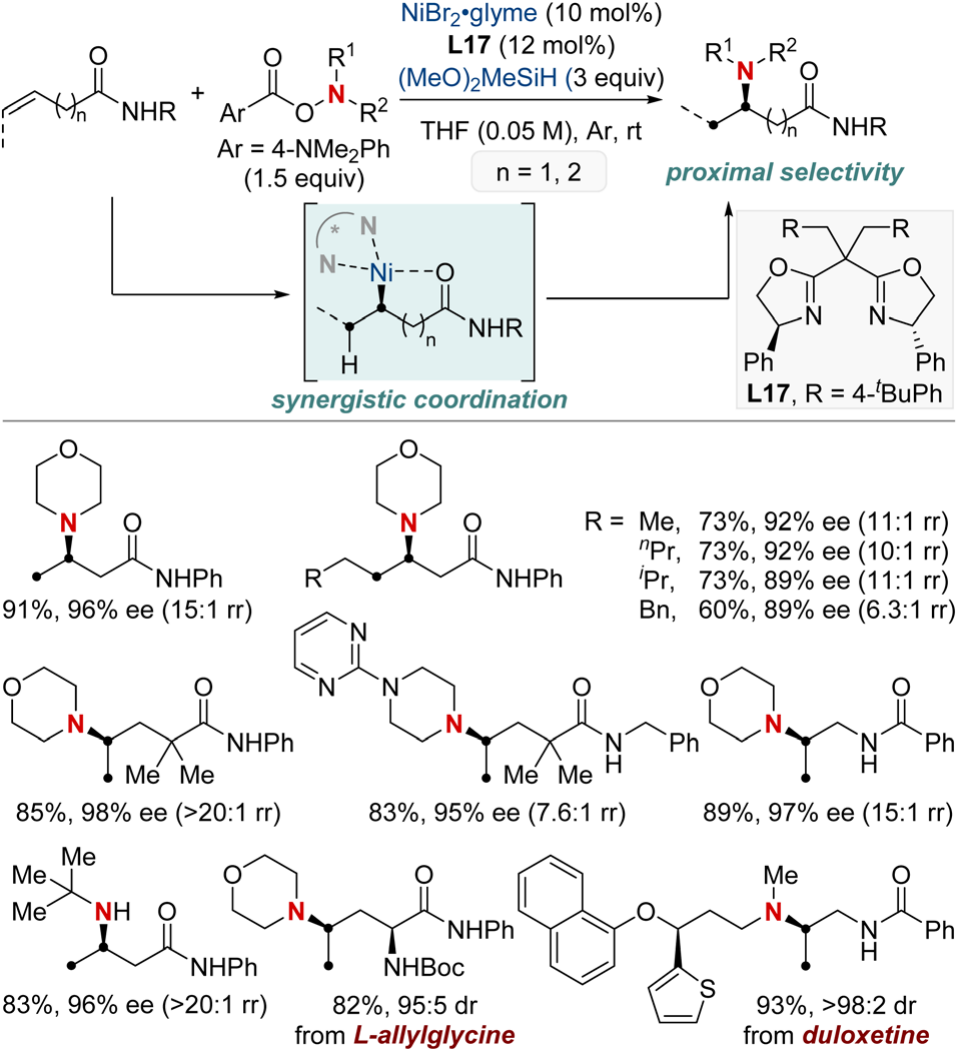
NiH-catalyzed asymmetric hydroamination of unactivated alkenes.

In 2022, the Hong group confronted a complex challenge in synthetic chemistry: the dual pursuit of enantio- and regio-selective hydroamination of relatively uncharted, unactivated alkenes (Scheme 20).^80^ Their pioneering approach revolved around a Ni-catalyzed hydroamination, directed by expertly designed chiral ligands. This approach targeted readily accessible unactivated alkenes, especially those with weakly coordinating functionalities such as native amides or esters. Their methodology showcased impressive versatility, accommodating both terminal and internal unactivated alkenes while maintaining compatibility with a broad spectrum of amine coupling partners. The mildness of the reaction conditions positioned their technique as an ideal choice for the late-stage diversification of complex molecules. Such versatility enabled the efficient synthesis of enantioenriched β- or γ-amino acid derivatives, as well as 1,2- or 1,3-diamines in a modular fashion. Further investigation into the mechanism underscored the significance of a chiral bisoxazoline-ligated Ni complex. Coupled with a carefully selected carbonyl directing group, this setup proved instrumental in guiding the sought-after enantio- and regioselective NiH insertion into the alkenes.

**Scheme 21:**
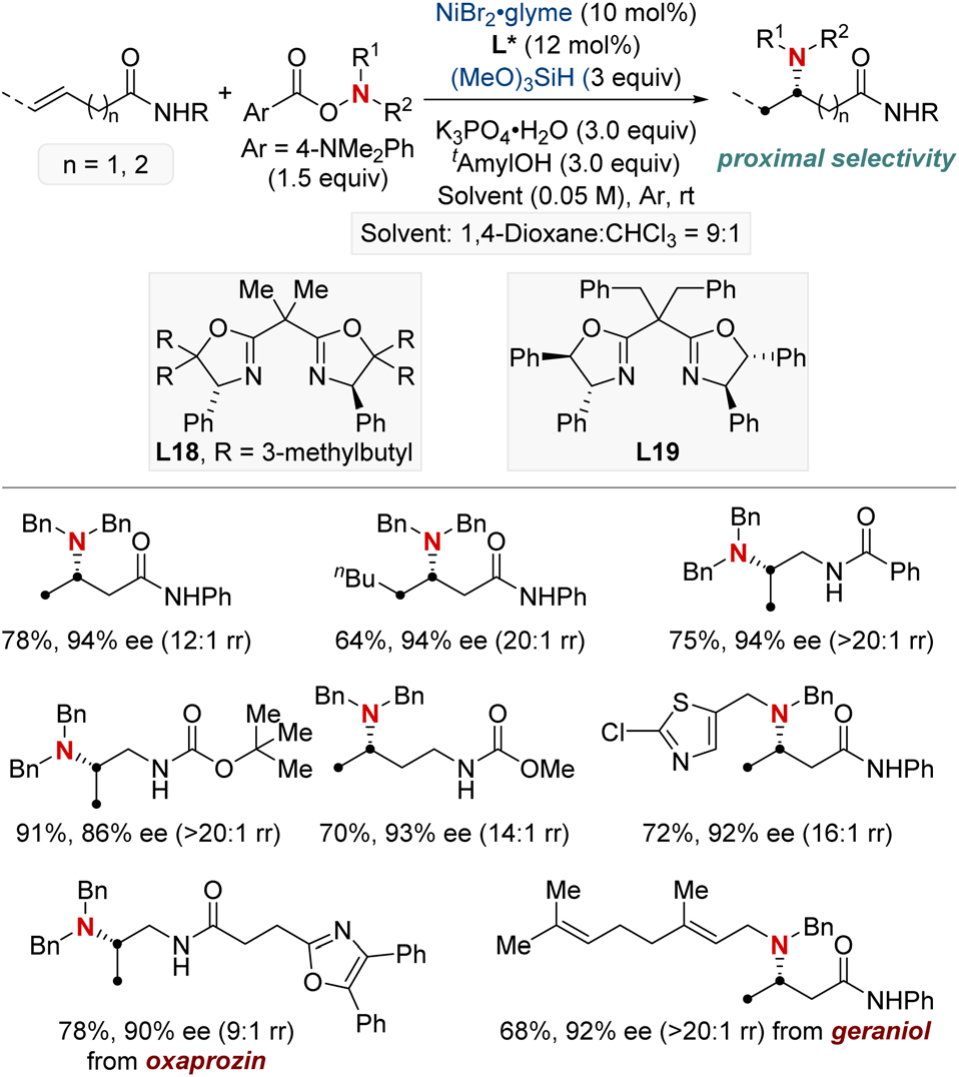
NiH-catalyzed asymmetric hydroamination of unactivated alkenes.

On a related note, the Shu group developed a Ni-catalyzed asymmetric hydroamination protocol employing weakly coordinating amide groups (Scheme 21).^81^ This method facilitated the efficient synthesis of enantioenriched amino acid derivatives and diamines featuring chiral α-branched aliphatic amine motifs. Demonstrating broad adaptability, their method embraced both terminal and internal unactivated alkenes, producing an assortment of enantioenriched amines characterized by varied substitution patterns. Furthermore, in-depth kinetic studies revealed that the nickel pre-catalyst and silane exhibited first-order dependence, highlighting the potential involvement of NiH regeneration in the turnover-limiting step. This discovery not only deepens the understanding of the reaction mechanism of hydoramination but also sets the stage for pioneering advances in the design of new metal hydride chemistry.

**Scheme 22:**
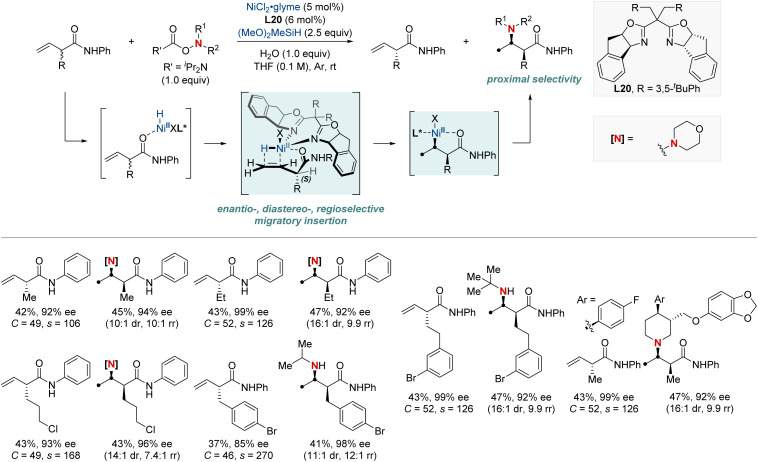
NiH-catalyzed enantio-, diastereo-, regioselective hydroamination enabling kinetic resolution.

In 2023, the Hong group marked a pivotal milestone in asymmetric hydroamination, employing kinetic resolution, which extracts enantioenriched compounds from racemic mixtures (Scheme 22).^82^ Their focus sharpened on a nickel-catalyzed kinetic resolution tailored for racemic α-substituted unconjugated carbonyl alkenes. This meticulously engineered method harmoniously combined enantioselectivity, diastereoselectivity, and regioselectivity in hydroamination. The result was the proficient creation of chiral α-substituted butenamides and *syn*-β^2,3^-amino acid derivatives. The hallmark of their achievement lay in the remarkable enantiomeric purity, with an exemplary enantiomeric excess (ee) of up to 99%. Augmenting their accomplishment was a striking selectivity factor that eclipsed >684. At the heart of this kinetic resolution's success was the chiral nickel complex; its intricate design was essential for achieving excellent resolution and enantioselective formation of C–N bonds.

**Scheme 23:**
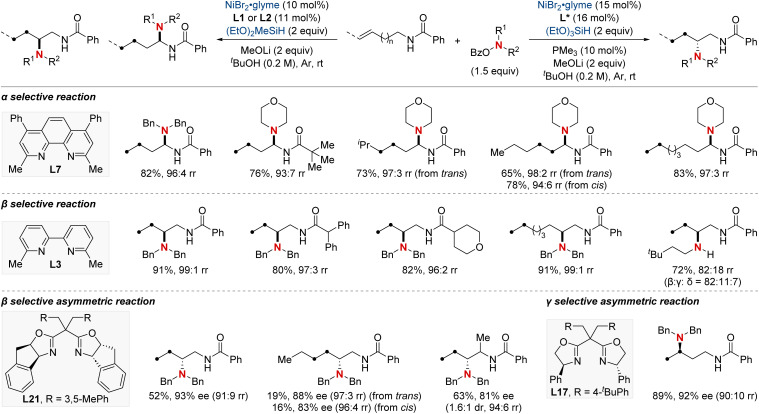
NiH-catalyzed regiodivergent migratory hydroamination and asymmetric hydroamination.

In 2023, the Wang group undertook a complex challenge, harnessing the combined potential of ligands and directing groups to advance transition-metal-catalyzed remote hydrofunctionalization of alkenes (Scheme 23).^83^ This research focused intently on optimizing the site-selective NiH-catalyzed hydroamination of unactivated alkenes, especially those bearing weakly coordinating amide groups. A cornerstone of their approach was the utilization of readily available bidentate nitrogen-containing ligands. This tactical choice enabled the streamlined synthesis of 1,1-, 1,2-, and 1,3-diamines from consistent substrates with outstanding regioselectivity. Additionally, their versatile methodology integrated a range of *O*-benzoyl hydroxylamine electrophiles, guiding them either *via* nickel chain-walking pathways or more straightforward techniques. By establishing these position-specific methods, the group set the foundation for the enantioselective crafting of the sought-after 1,2-diamines (through distant aliphatic C–H amination) and 1,3-diamines. This accomplishment underscores the profound implications of their technique in the evolving realm of chemical synthesis.

**Scheme 24:**
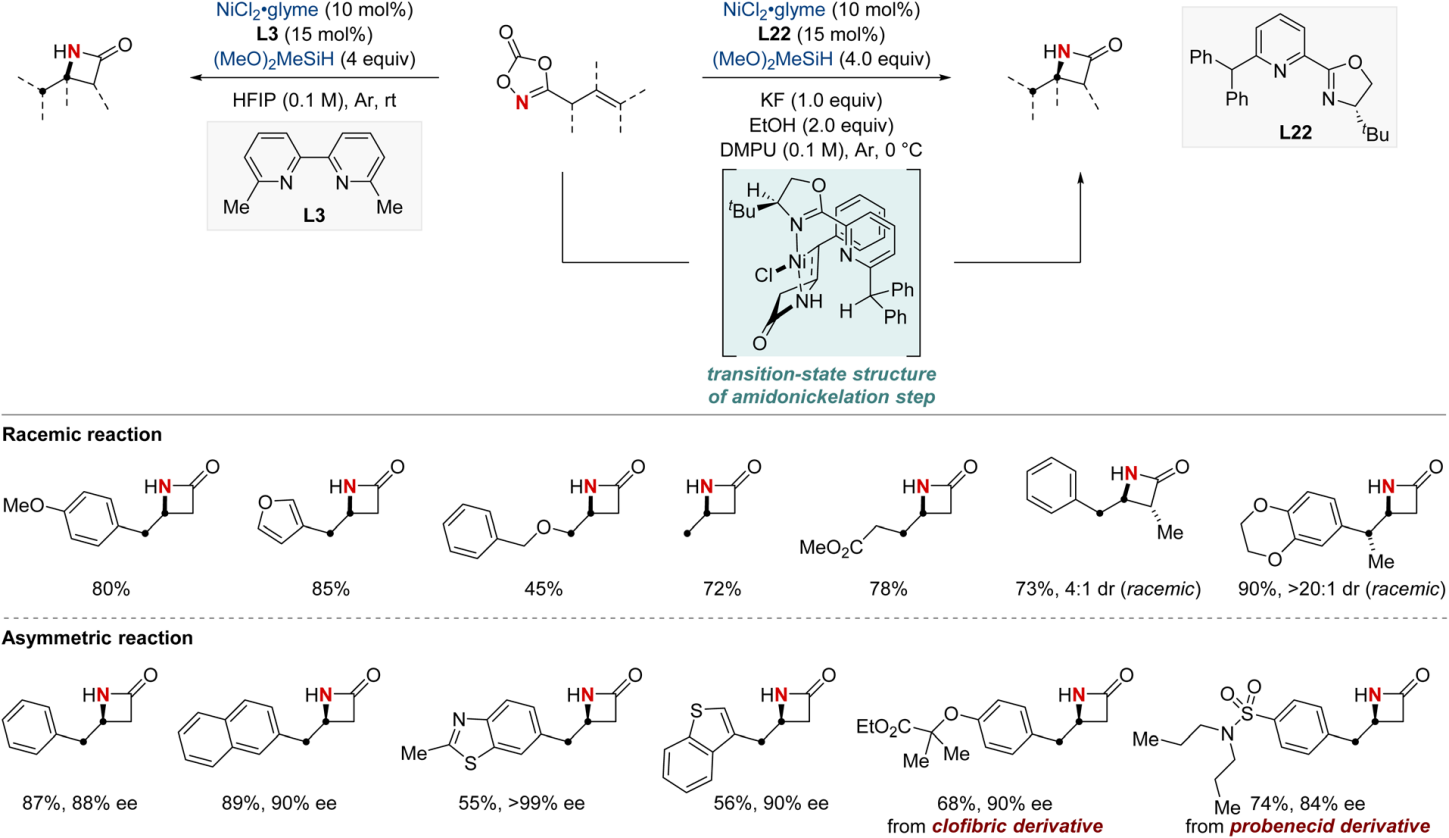
NiH-catalyzed intramolecular asymmetric hydroamidation.

In 2023, the Chang group achieved a significant breakthrough in developing synthetic techniques for the synthesis of enantioenriched β-lactams—compounds renowned for their prevalence in bioactive molecules.^84^ Confronting the longstanding issue of regioselectivity in the intramolecular hydroamidation of β,γ-unsaturated amides, the group unveiled a pioneering NiH-catalyzed strategy that harnesses easily obtainable alkenyl dioxazolone derivatives. Breaking away from conventional NiH frameworks, their method utilized a unique mechanism initiated by N-activation. This not only enabled proximal C–N bond formation with exemplary regioselectivity but also remained indifferent to variations in substituent electronic attributes. Together with their valuable mechanistic insights, this advancement paved the way for a streamlined synthesis of enantioenriched β-lactams. The merit of this protocol is underscored by the efficient synthesis of a key β-lactam intermediate, pivotal to the total synthesis of haouamine B. The lactam compound was prepared within a concise nine-step synthetic process, an improvement over the previously established 14-step procedure. This achievement marks a substantial advancement in synthetic methodology, demonstrating the effectiveness and efficiency of the novel approach adopted in this study (Scheme 24).

## 5. Conclusions and outlook

C–N bond formation stands as a cornerstone of organic synthesis, with hydroamination distinguishing itself as an incredibly versatile strategy for the synthesis of numerous essential amine molecules. The adoption of this approach has marked a transformative move towards greater efficiency and sustainability, turning the traditional thermoneutral approach into a more energetically favorable route. The rise of NiH catalysis in this domain is unequivocal, with its application in regio- and enantioselective hydroamination and hydroamidation reactions positioning it as a significant and promising player for the future. This review illustrates recent advancements in NiH-catalyzed functionalization, categorizing them into distinct sections: regioselective C–N bond formation across alkenes and alkynes and asymmetric C–N bond synthesis involving alkenes. Current research emphasizes the adaptability of NiH-catalyzed hydroamination and hydroamidation, showcasing its capacity for the efficient synthesis of an array of amine or amide derivatives from readily available olefinic feedstocks. Beyond cost considerations, where nickel presents an advantage over other precious metals, its inherent catalytic strengths are of notable significance. Especially in the realm of metal hydrides, nickel's unique attributes become evident in its applications across alkenes and alkynes. Innovations like rapid chainwalking and meticulous coordination amplify its effectiveness to drive precise and desired outcomes. The pursuit of chiral molecule production *via* asymmetric induction exemplifies the vast potential in this domain.

Despite significant advancements in the field, challenges in asymmetric hydroamination and hydroamidation of alkenes still remain. Innovations in ligand design, control of divergent pathways, and adherence to green chemistry principles are set to transform organic synthesis methodologies. The development of enhanced ligands and catalysts is anticipated to play a crucial role in driving innovative reactivity exploration. In addition, existing methods often necessitate specific reaction conditions and rely heavily on expensive ligands and catalysts, posing scalability and environmental concerns. Therefore, a primary hurdle is the development of efficient chiral ligands and catalysts that are both cost-effective and versatile enough for intricate molecular syntheses and large-scale industrial applications. In this context, future catalysts must prioritize efficiency by using fewer reactants, achieving faster reaction rates, and providing exceptional regio- and enantioselectivities. Additionally, these next-generation catalysts should exhibit adaptability to various functional groups, enabling the handling of complex, late-stage modifications while maintaining stability under ambient conditions through improved durability. There is a pressing need to broaden the spectrum of suitable alkenes and electrophiles developing finely tuned highly reactive amination reagents. In aiming high, the field anticipates advancements that will generate multiple chiral centers, introduce heteroatoms, and facilitate the synthesis of quaternary amine structures, opening up new possibilities for chemical reactions.

Moreover, the exploration of regiodivergent reactions, where catalyst systems yield different regioisomers from the same reactants, is particularly intriguing. This strategy transcends regioselective limitations, offering diverse product possibilities under controlled conditions. The potential for ligand-controlled regiodivergent reactions is substantial, as adjusting ligand structures could allow chemists to steer NiH-catalyzed reactions towards specific regioisomers, paving the way for the synthesis of complex molecules with varied structural frameworks. External factors like reaction temperature and time are also critical in determining regioselectivity and enantioselectivity in NiH-catalyzed reactions. Investigating these variables could lead to more adaptable and efficient synthetic methods, broadening the utility of NiH catalysis.

Finally, integrating green chemistry principles such as renewable resource utilization, waste minimization, energy efficiency, and cleaner production techniques, NiH catalysis can make a substantial contribution to more sustainable chemical processes. This involves moving away from traditional stoichiometric silanes towards greener reductants. The use of environmentally benign reductants not only diminishes the environmental impact of these reactions but also aligns with green chemistry ideals. Exploring bio-based reductants or harnessing renewable resources presents sustainable alternatives for NiH generation. A groundbreaking strategy is adopting photocatalysis in NiH generation. Leveraging light, an abundant and clean energy source, to facilitate NiH formation marks a significant advancement in green methodologies. Photocatalysis could potentially reduce energy consumption and curb reliance on non-renewable energy sources. Implementing immobilized chiral catalysts in NiH catalysis also promotes greener chemistry. These catalysts, which enable easy separation and potential reusability, help to decrease the need for excessive reagents and thereby minimize waste. Developing durable, recyclable catalysts that retain high efficacy through multiple uses is essential. This method addresses reaction scalability and markedly reduces environmental impact by lowering waste and resource use. Moreover, employing real-time monitoring and flow chemistry techniques can optimize reaction conditions for maximum efficiency while reducing waste and harmful by-products. These methods not only bolster the sustainability of the process but also aid in refining catalytic systems for enhanced efficiency.

In conclusion, the collected insights within this review highlight the substantial advancements made in the field of NiH-catalyzed hydroamination in organic chemistry. With these insights, our intention is to pave the path for researchers, emphasizing the opportunities in C–N bond formation and serving as inspiration for continued innovation and significant milestones in the realm of organic synthesis.

## Author contributions

C. L., H. K. and S. H. conceived the idea and contributed to the writing, review and editing of the manuscript. All authors have given approval to the final version of the manuscript.

## Conflicts of interest

There are no conflicts to declare.

## Supplementary Material
